# Long-term effects of payment for performance on maternal and child health outcomes: evidence from Tanzania

**DOI:** 10.1136/bmjgh-2021-006409

**Published:** 2021-12-16

**Authors:** Josephine Borghi, Peter Binyaruka, Iddy Mayumana, Siri Lange, Vincent Somville, Ottar Maestad

**Affiliations:** 1 Department of Global Health and Development, London School of Hygiene & Tropical Medicine, London, UK; 2 Ifakara Health Institute, Dar es Salaam, Tanzania, United Republic of; 3 Chr Michelsen Institute, Bergen, Norway; 4 Ifakara Health Institute, Ifakara, Morogoro, Tanzania, United Republic of; 5 Department of Health Promotion and Development, University of Bergen, Bergen, Hordaland, Norway; 6 NHH Norwegian School of Economics, Bergen, Norway

**Keywords:** maternal health, health economics, health systems evaluation

## Abstract

**Background:**

The success of payment for performance (P4P) schemes relies on their ability to generate sustainable changes in the behaviour of healthcare providers. This paper examines short-term and longer-term effects of P4P in Tanzania and the reasons for these changes.

**Methods:**

We conducted a controlled before and after study and an embedded process evaluation. Three rounds of facility, patient and household survey data (at baseline, after 13 months and at 36 months) measured programme effects in seven intervention districts and four comparison districts. We used linear difference-in-difference regression analysis to determine programme effects, and differential effects over time. Four rounds of qualitative data examined evolution in programme design, implementation and mechanisms of change.

**Results:**

Programme effects on the rate of institutional deliveries and antimalarial treatment during antenatal care reduced overtime, with stock out rates of antimalarials increasing over time to baseline levels. P4P led to sustained improvements in kindness during deliveries, with a wider set of improvements in patient experience of care in the longer term. A change in programme management and funding delayed incentive payments affecting performance on some indicators. The verification system became more integrated within routine systems over time, reducing the time burden on managers and health workers. Ongoing financial autonomy and supervision sustained motivational effects in those aspects of care giving not reliant on funding.

**Conclusion:**

Our study adds to limited and mixed evidence documenting how P4P effects evolve over time. Our findings highlight the importance of undertaking ongoing assessment of effects over time.

**Key questions**

**What is already known?**

- An increasing number of studies have examined the impact of payment for performance (P4P) schemes at one point in time, reporting positive effects on some targeted outcomes.
- Evidence from high-income settings suggests P4P effects diminish over time, but effects are more likely to be sustained in low-performance areas.
- Evidence from low-income settings is limited and mixed.
- We know little about why effects change over time, though there are varying hypotheses as to how and why they might change.

**What are the new findings?**

- The effects of the programme on the rate of institutional deliveries and antimalaria treatment during antenatal care reduced overtime, with stock out rates of intermittent presumptive treatment increasing over time to baseline levels after an initial reduction.
- There was evidence of sustained improvements in kindness during deliveries, and indications of a wider set of improvements in patient experience of delivery care in the longer term.
- It took time for health workers to fully understand and grasp the programme and the verification system became more integrated within routine systems over time, reducing the time impact of the programme on managers and health workers.

**What do the new findings imply?**

- Our findings highlight the importance of not just evaluating the effects of P4P at one point in time, but in undertaking ongoing assessment of effects over time.
- It is clearly important for evaluators to monitor changes in programme design and implementation and how this is related to outcomes, especially as schemes go from pilot to scale, and are taken over by government.
- Results demonstrate the limitations of conventional evaluations of cause and effect, and the need to embrace a complex adaptive systems approach to understanding health systems and their response to P4P.

## Introduction

Coverage and quality of essential and effective health services in low-income and middle-income countries (LMIC) remains inadequate, limiting gains in health outcomes.^1 2^ Over the last 10 years, many LMICs have introduced performance-based incentives to strengthen health systems and enhance the coverage and quality of health services.^3^ Payment for performance (P4P) schemes consist of payments to healthcare providers contingent on the improvement of predefined performance indicators, though their design varies substantially across settings.^4^ In low-income countries, part of the payment is paid directly to health workers, and part of the payment is paid to the facility for investment in improved service delivery, with healthcare managers often receiving payments based on the performance of health facilities under their jurisdiction.^4^

An increasing number of studies have examined the impact of P4P schemes at one point in time, reporting positive effects on some targeted outcomes.^5–9^ However, there has been less attention to documenting whether and how the effects of P4P programmes vary over time. This paper contributes to filling this gap by comparing the short-term (after 13 months) and longer-term (36 months) impact of P4P in Tanzania.

There are a number of reasons why the impacts of P4P may vary over time, with temporal responses depending on programme design and actor response to this. In schemes rewarding based on threshold targets, goal gradient theory suggests effort will increase as agents move closer to the goal,^10^ and cease once the threshold is reached,^11 12^ with multiple threshold targets being expected to encourage sustained effort.^13^ Like many complex interventions, the design of P4P programmes is not static, and adaptations are commonplace during implementation,^14^and can result in changing effects over time. Further, actor response to incentives may not be constant. When incentives are tied to tasks involving complex processes, experience and learning may be a prerequisite for improved performance—with actors taking time to understand the scheme, develop strategies and systems to improve performance.^3 15^ As a result, changes in behaviour may not be observed immediately.^16^ It may also take time for providers to develop trust that performance payments will be made, especially in fragile states with weak accountability systems.^17^ Finally, there may be a lag between patient recognition of enhanced health system responsiveness linked to incentives, and the adjustment of care-seeking behaviour within the community.^18^ In contrast, self-determination theory posits that P4P schemes may result in weaker effects over time, in so far as monetary rewards *crowd out* intrinsic work motivation.^19 20^ Equally, results may reduce over time due to *reduced salience* of the scheme as incentives become normalised.

Therefore, it is difficult to hypothesise the temporal variation in programme effects. Empirical studies are needed to improve our understanding of how effects vary over time in response to programme design, implementation and contextual factors.

Numerous studies from high-income countries with good routine health information systems have examined the effects of P4P over time. In the United States, the United Kingdom and Australia, 21 22,23 P4P effects were found to diminish over time, with suggestions that where baseline performance is lower there is more potential for longer-term effects to be sustained.^23^ Studies in Taiwan have found sustained effects on some services^24^ and sustained but reduced effects over time for others.^25^ In low-income settings, a few studies have examined how incentive effects vary over time, with mixed effects reported in Mozambique^15^ and Zimbabwe.^26^ However, these studies did not explore the reasons for changes in the effectiveness of P4P over time. Thus, there is a need for more evidence from LMICs to better understand the dynamic temporal effects of P4P schemes, and how and under which circumstances changes in effects occur.

This paper presents an extension of our previous evaluation of P4P in Tanzania after 13 months^6^ where we reported positive programme effects on two out of eight incentivised service indicators, and no effects on other indicators^6^; identified positive programme effects on the availability of drugs and medical supplies^27^; and considered the heterogeneity of effects across population^28^ and provider subgroups.^29^ Here, we consider the longer-term effects of the programme over an additional 23 months to examine whether there has been a broadening of effects over time, and an enhancement or reduction in initial achievements. In parallel, we consider whether there were changes in programme design, implementation and mechanisms that might explain variations in outcome over time.

### The P4P scheme

A P4P scheme was introduced in Pwani region in 2011 by the Ministry of Health and Social Welfare (MOHSW) with funding from the Norwegian Ministry of Foreign Affairs. The scheme provided financial payments to health facilities and district and regional health managers based on achievement of predefined targets for coverage of maternal and child health services (eg, institutional delivery; postnatal care (PNC) within 7 days of delivery) and content of care (eg, two doses of intermittent presumptive treatment (IPT) for malaria during antenatal care (ANC)) (table 1). The extensive use of service coverage indicators within the scheme distinguishes it from the fee-for-service schemes which are more commonly applied in other low-income settings. All except one of the eight incentivised service coverage indicators involved multiple thresholds based on performance in the previous cycle. One indicator (IPT provision during ANC) involved a single absolute threshold target. Performance was measured through the Health Management Information System (HMIS) every 6 months.

**Table 1:** Scheme design

Performance indicators	Method	Baseline performance (previous cycle)				
		0%–20%	21%–40%	41%–70%	71%–85%	85%+*
Service coverage indicators						
Institutional delivery rate	Percentage point increase	15	10	5	5	Maintain
% of mothers attending a facility within 7 days of delivery.	Percentage point increase	15	10	5	5	Maintain
% of women using long-term contraceptives	Percentage point increase	20	15	10	Maintain above 71	Maintain
% children under 1 year received measles vaccine	Overall result	50	65	75	80+*	Maintain
% children under 1 year received Penta 3	Overall result	50	65	75	80+	Maintain
Content of care indicators						
% ANC clients on IPT2	Overall result	80	80	80	80+	Maintain above 80
% HIV+ ANC clients on Antiretroviral Therapy	Overall result	40	60	75	75+	Maintain
Polio vaccine (OPV0) at birth	Overall result	60	75	80	80+	Maintain

Source: The United Republic of Tanzania, Ministry of Health and Social Welfare, 2011. The Coast Region Pay for Performance (P4P) Pilot: Design Document.

*80+ : 80% or more.

ANC, antenatal care; IPT, intermittent presumptive treatment.

Performance data were verified each 6-month cycle by national, regional and district stakeholders by comparing reported data to facility registers. The performance payments were shared between health workers (75% of the total) and the facility for investment in service delivery improvements (25%). The allocation of payments across health workers was at the discretion of the facility. To receive any payment, facilities had to meet at least 75% of the target, with 100% achievement being required for full payment. The maximum payout per cycle was US$820 for dispensaries; US$3220 for health centres; and US$6790 for hospitals. The health worker component is in the order of 10% of the salary for the maximum payout and average number of staff. At the district and regional level, managers were incentivised based on performance of facilities in their areas, together with drug availability and timely submission of HMIS reports, receiving up to US$3000 per cycle.

During the period 2011–2013 the implementation of P4P was supported by the Clinton Health Access Initiative (CHAI) who assisted in the calculation of payouts, participated in performance feedback meetings every cycle with district managers and healthcare workers and in data verification activities. From January 2014 Norwegian funding could no longer support bonus payments, with funding for CHAI ending in June 2014. Thereafter the MOHSW managed the scheme with the World Bank Health Innovation Trust Fund supporting bonus payments. However, agreement between the government of Tanzania and the World Bank was not finalised until March 2015, resulting in the delay of P4P payments for two cycles.^30^

## Methods

### Study design

This is a mixed method study which was guided by a theory of change including a quantitative impact assessment and a qualitative process evaluation. The impact assessment used a controlled before and after study design. Data for the impact assessment were collected at three points in time, just before the first incentive payments in January 2012, 13 months later (referred to as short term), and 36 months later (referred to as long term).^32^ The minimum time necessary to detect initial programme effect was deemed to be 13 months and 36 months was selected for the third round as it was just before the end of the pilot programme before its transition to a Results Based Financing scheme which was gradually rolled out nationally. Data were collected in all seven intervention districts and four comparison districts from neighbouring regions (Morogoro and Lindi) that were similar in relation to poverty and literacy rates, the rate of institutional deliveries, infant mortality, population per health facility and the number of children under 1 year of age per capita. Care was also taken to avoid districts where programmes were underway to improve maternal and child health, which could confound results.

Process evaluation data about programme design, implementation of the programme and change mechanisms were collected over three rounds in the short term (December 2011–March 2013) and one round in February 2015 to examine longer-term changes.

A theory of change guided the evaluation and was developed with reference to existing literature and based on discussion with national stakeholders. It is described in the study protocol,^31^ but a summary follows. P4P is expected to improve the quality of care of targeted services through an increase in health worker and manager motivation to obtain bonus payments, which is assumed to increase service coverage. If motivated to achieve targets, health workers might make services more accessible by reducing waiting time, ensuring drugs are available at the facility, following clinical guidelines that may lengthen consultations, reducing user charges and being more friendly and attentive to patients, resulting in greater patient satisfaction. Unintended consequences that could result from the P4P scheme include reductions in the use and quality of unincentivised health services. Furthermore, the quality of targeted services may decline over time, if health workers become overburdened and utilisation increases beyond available facility capacity.

### Data sources

#### Quantitative

We sampled 75 facilities from Pwani region and the same number from comparison districts, including hospitals (n=6), health centres (n=16) and dispensaries (n=53) in each arm. Comparison facilities had similar levels of outpatient care visits and staffing levels to intervention facilities. Facilities were sampled to achieve district representation, with 46% of all facilities in Pwani region being included in the sample. No sample size calculation was therefore carried out. We collected data through surveys of facilities, patient exit interviews and interviews at household level with women who had given birth in the past 12 months. The full sampling strategy is outlined in the study protocol^31^ but a summary follows, with more details in online supplemental appendix 1.

10.1136/bmjgh-2021-006409.supp1 Supplementary data

A total of 1500 women were sampled within the catchment areas of facilities in each arm and each round. The survey measured coverage of targeted maternal and child health services, satisfaction with delivery care, user costs for three of the targeted services and household socioeconomic characteristics.^32^ Seven hundred and fifty patient exit interviews were conducted in each arm per round with patients attending ANC or PNC, and women with children under 1 year of age coming for a preventive check-up or an immunisation. Sample sizes for the women and patient surveys are reported in online supplemental appendix 1. We collected data on process quality for incentivised (ANC and PNC, delivery and immunisation services) and non-incentivised services (outpatient visits for children under 5 presenting with fever, cough or diarrhoea). We measured provider adherence to clinical care guidelines for ANC (a 21-item index); waiting time (in minutes); kindness during delivery (using a 10-point scale) and patient satisfaction with provider–client interactions (an index of 13–19 items adapted from^33^). Facility surveys gathered data on monthly numbers of outpatient visits by age (under and over 5 years of age) from patient registers for the period January 2010 to December 2014.^32^ Facility surveys also gathered data on structural quality of care in terms of the availability (on the day of the survey) and stock out (in prior 90 days) of essential drugs (n=37), medical supplies (n=11) and equipment (n=16). We also looked at the availability/stock out of delivery care drugs (n=8), antimalarials (n=2) and antiretrovirals (n=7) as being related to incentivised services.^32^ For each of these groupings, we generated composite scores based on an unweighted mean score across items in the group, which can be interpreted as the mean percentage availability/stock‐out rate within the grouping across facilities.

#### Qualitative

The findings from the first three rounds of process evaluation data covering short-term implementation of P4P have been presented elsewhere.^34^ In this paper we focus on the findings from the most recent round of data collection (February and March 2015) which covers implementation in 2014. These findings were contrasted with the earlier process evaluation findings to identify implementation changes over time.

In this round, in-depth interviews were done in 24 facilities from two intervention districts (Bagamoyo and Kisarawe), including 19 dispensaries, 4 health centres and 1 hospital. Twenty-one facilities were public, the remainder were faith based/not for profit. Apart from the hospital, all the facilities were located in rural areas. In-depth interviews were done with the in-charge and/or health workers responsible for maternal and child health services and lasted about an hour. Interviews were also conducted with one or more district managers (Council Health Management Team) from four districts (Bagamoyo, Kibaha, Kisarawe and Mkuranga). The main purpose of the interviews was to understand health worker perceptions and response to the programme, including the use of bonus payments and strategies for achieving targets, and whether and how this changed over time. Sampled facilities differed in terms of remoteness, staffing numbers and characteristics. Towards the end of data collection, no new themes emerged. Two researchers (IM and SL) conducted all the interviews in Swahili. All interviews were recorded and later transcribed and translated into English.

### Data analysis

#### Quantitative

We used a linear difference-in-difference regression model with facility and year fixed effects to determine the effects of P4P over time and the difference between the short-term and the longer-term effects. To determine the short-term effects of the programme (2012–2013), we compared the changes in outcomes at 13 months compared with the baseline in P4P facilities to the change in facilities without P4P. To determine the longer-term effects of the programme (2012–2015), we compared the change in outcomes at 36 months to the baseline in P4P facilities to the change in facilities without P4P. We estimated separate effects for the short-term and long-term periods by including terms for the interaction between the intervention group and each of the two post-implementation periods (online supplemental appendix 2). We also estimated the difference between the short-term and long-term effects (online supplemental appendix 2). In the analysis of women’s and patients’ outcomes, we controlled for individual characteristics (education, religion, marital status, occupation, age, number of pregnancies) and household characteristics (insurance status, number of household members, household head education and wealth based on ownership of household assets and housing particulars). Standard errors were clustered at the facility level, or the facility catchment area.

We further estimated the heterogeneity of P4P effects across local area characteristics (wealth status, rural/urban location) and characteristics of facilities (level of care, ownership, baseline performance, above and below the median performance for deliveries and IPT during ANC)^29^ by including a three-way interaction term and controlling for time-varying facility-level covariates (availability of electricity and water supply, and the mean wealth index for households sampled in the catchment area of the facility) as potential confounding factors (online supplemental appendix 2).

The identifying assumption of the difference-in-difference approach is that the outcomes between study arms would have followed parallel trends in the absence of the intervention. We previously verified that trends in a number of outcomes at the household and facility levels were similar between the intervention and comparison areas prior to the introduction of P4P^6^ (online supplemental appendix 3). We also verified preintervention trends were parallel in facility service utilisation levels based on patient registers.^6^

The outcomes considered are those reported previously^6^: notably the eight incentivised indicators as well as indicators which could be indirectly affected by incentives (coverage of ANC and PNC) and non-targeted services (outpatient visits). We examined programme effects on quality of care measures, including effects on the availability and stock out of essential drug and supplies^27^ and on the probability of paying and costs of key maternal care services, and related gifts.

To take the multiple testing into account, we correct the p values by hypothesis using the Bonferroni correction (the p value threshold for statistical significance at the 5% level becomes equal to 0.05/(number of tests)). The grouping of the tests by hypothesis is listed in online supplemental appendix 4.

We present descriptive analyses of health worker and facility survey data in rounds 2 and 3, to determine implementation reach.

All statistical analyses were done with Stata (V.16).

#### Qualitative

The data were double coded using NVivo V.9 software, employing an inductive framework relating to the core research questions, comparing and contrasting perceptions and strategies employed early on and later in the programme, together with design adaptations and challenges experienced over time.

### Patient and public involvement

Patients were not directly involved in the design or dissemination of the study.

### Data

The quantitative data for this paper are made available through Zenodo, DOI: 10.5281/zenodo.5636645, https://zenodo.org/record/5636646%23.YanUmtnMK3I

## Results

### Impact evaluation findings

At baseline, coverage of institutional deliveries was over 84% (table 2). Two vaccination indicators (polio vaccine at birth and three doses of pentavalent vaccine) also had a baseline coverage of >75%. Baseline coverage of other incentivised services varied between 22% and 51%. Baseline coverage levels for incentivised indicators were generally similar between intervention and comparison groups (table 2). However, baseline coverage of IPT2 and four or more ANC visits was higher in comparison areas, 56.7% versus 49.5% (p=0.005) and 65.0% versus 71.2% (p=0.02), respectively. Baseline coverage of PNC within 7 days was higher in the intervention area, 21.5% versus 16.9% (p=0.043).

**Table 2:** Service use before and after the introduction of payment for performance

	Intervention			Comparison		
	Baseline	Short term	Long term	Baseline (p value)	Short term	Long term
**Targeted services**						
At least two doses of IPT during ANC (%)	49.5	72.9	67.5	56.7 (0.005)	69.2	68.4
HIV treatment during ANC (%)	7.8	6.1	13.1	6.8 (0.527)	6.2	8.8
Institutional delivery rate (%)	84.7	89.2	92.2	86.8 (0.350)	83.1	89.4
Polio vaccine at birth (%)	77.4	79.1	80.0	78.5 (0.668)	74.4	77.3
Measles (%)	51.4	44.3	25.7	53.3 (0.654)	34.2	33.7
Penta 3 doses (%)	76.4	76.6	74.9	79.9 (0.243)	74.4	74.0
Postnatal care in facility <7 days (%)	21.5	19.6	24.9	16.9 (0.043)	13.8	18.3
Use of long-term family planning (%)	36.7	26.4	22.1	39.2 (0.384)	30.7	22.1
**Non-targeted aspects of targeted services**						
Any ANC visit (%)	97.2	99.3	99.9	99.9 (0.001)	98.9	99.7
Four or more ANC visits (%)	65.0	64.8	61.5	71.2 (0.020)	67.9	67.3
Postnatal care in facility <2 months (%)	27.7	21.6	34.3	23.4 (0.120)	18.6	30.8
**Non-targeted services**						
Outpatient visits per facility per month >5 years	359.5	334.1	393.6	287.3 (0.000)	291.6	260.9
Outpatient visits per facility per month >5 years, dispensaries	276.8	190.6	178.3	235.4 (0.006)	236.3	165.1
Outpatient visits per facility per month <5 years	223.9	164.1	182.1	193.7 (0.011)	185.5	159.8
Outpatient visits per facility per month <5 years, dispensaries	164.8	93.2	124.4	172.6 (0.441)	160.2	111.7

P values in parenthesis are for the baseline differences between intervention and comparison areas.

ANC, antenatal care; IPT, intermittent presumptive treatment.

In the short term, P4P affected two out of eight indicators incentivised at the facility level; a 10.3 percentage point increase in the provision of IPT during ANC (p=0.001), and an 8.2 percentage point increase in the rate of institutional deliveries (p=0.001) (tables 2 and 3 and online supplemental appendix 5). These short-term effects are robust to correcting for multiple testing (at the 5% level of significance, the Bonferroni threshold for the p values is equal to 0.0055) (online supplemental appendix 4). In the longer run, there was a smaller effect on institutional deliveries (4.9 percentage points (p=0.018), but the decline was not statistically significant (3.2 percentage points p=0.114). The estimated effect on IPT coverage during ANC was also smaller in the longer term, and only borderline significant (5.6 percentage points (p=0.097)). While no short or long term effects were identified, there was an important reduction in measles immunisation coverage between the short and the longer term by 15.6 percentage points (p=0.013, not significant with the Bonferroni correction) =, and an increase in coverage of HIV treatment during ANC by 4.3 percentage points (p=0.085). There was no longer-term impact on any of the other incentivised indicators that did not change in the short run.

**Table 3:** Direct and indirect effect of payment for performance on the use of targeted services in the short and long term (results from the difference-in-difference analysis)

Outcome variables	N	Short-term effect		Difference between short and long term		Long-term effect	
		Beta* (95% CI)	P value	Beta* (95% CI)	P value	Beta* (95% CI)	P value
**Targeted services**							
At least two doses of IPT during ANC (%)	7362	10.3 (4.4 to 16.1)	0.001	−4.7 (−10.8 to 1.4)	0.129	5.6 (−1.0 to 12.4)	0.097
HIV treatment during ANC (%)	8472	−0.3 (−4.2 to 3.7)	0.893	4.3 (−0.6 to 9.3)	0.085	4.0 (−1.5 to 9.5)	0.157
Institutional delivery rate (%)	8728	8.2 (3.6 to 12.8)	0.001	−3.2 (−7.1 to 0.8)	0.114	4.9 (0.8 to 8.9)	0.018
Institutional delivery rate (%) (public)	8728	6.5 (1.3 to 11.7)	0.015	−0.1 (−5.0 to 4.9)	0.982	6.3 (1.3 to 11.3)	0.015
Polio vaccine at birth (%)	8728	5.6 (−1.0 to 12.2)	0.093	−1.7 (−7.2 to 3.7)	0.527	4.0 (−2.5 to 10.5)	0.222
Measles† (%)	1782	9.6 (−2.5 to 21.6)	0.119	−15.6 (−27.8 to −3.4)	0.013	−6.5 (−18.4 to 5.4)	0.281
Penta 3 doses‡ (%)	3852	2.4 (−6.6 to 11.4)	0.597	0.9 (−7.9 to 9.8)	0.833	4.5 (−5.1 to 14.1)	0.359
Postnatal care in facility <7 days (%)	8726	0.6 (−5.0 to 6.3)	0.823	1.3 (−4.5 to 7.2)	0.648	1.9 (−4.0 to 7.8)	0.511
Use of any family planning (%)	8345	−0.7 (−7.4 to 6.0)	0.844	−4.6 (−0.1 to 9.4)	0.057	3.9 (−2.1 to 9.8)	0.202
**Non-targeted aspects of targeted services**							
Any ANC visit (%)	8723	3.3 (1.5 to 5.1)	0.000	−0.2 (−1.1 to 0.7)	0.650	3.1 (1.4 to 4.8)	0.000
Four or more ANC visits (%)	8653	3.9 (−2.7 to 10.4)	0.245	−2.5 (−8.1 to 3.2)	0.389	1.4 (−5.1 to 7.9)	0.672
Postnatal care in facility <2 months (%)	8726	−1.6 (−8.1 to 4.9)	0.625	0.4 (−7.1 to 7.9)	0.916	−1.2 (−8.8 to 6.3)	0.745

*The beta is the estimated intervention effect controlling for a year dummy, facility-fixed effects, individual-level and household characteristics.

†Among infants aged 9–11 months.

‡Among infants aged 6–11 months.

ANC, antenatal care; IPT, intermittent presumptive treatment.

We also considered the effect of P4P on services which were indirectly incentivised. In the short term, we found that P4P was associated with a significant increase in coverage of at least one ANC visit by 3 percentage points (p<0.001), which was sustained in the longer term (tables 2 and 3 and online supplemental appendix 5). This effect is also robust to the Bonferroni correction (threshold=0.017), online supplemental appendix 4. We examined the effect of P4P on unincentivised care and found no significant effect on outpatient department visits (OPD) overall (table 4). Among dispensaries, there was a short-term reduction in OPD (by 91 visits and 58 visits per month for over 5 year olds and under 5 year olds, respectively), but no programme effect on these outcomes in the longer term.

**Table 4:** Effect of payment for performance on the use of non-targeted services in the short and long term (results from the difference-in-difference analysis)

Outcome variables	Facilities	Short-term effect			Difference between short and long term			Long-term effect		
		N	Beta* (95% CI)	P value	N	Beta* (95% CI)	P value	N	Beta* (95% CI)	P value
Outpatient visits per facility month >5 years	96	3353	−15.8 (−101.1 to 69.5)	0.714	4798	162.3 (65.2 to 259.5)	0.001	5422	73.0 (−36.6 to 182.5)	0.189
Outpatient visits per facility month >5 years, dispensaries	69	2538	−90.8 (−156.5 to −25.2)	0.007	3570	71.8 (30.8 to 112.8)	0.001	4075	−50.5 (−116.6 to 15.7)	0.133
Outpatient visits per facility month <5 years	93	3247	−41.1 (−93.2 to 10.9)	0.120	4754	80.52 (33.1 to 127.9)	0.001	5278	−2.8 (−51.7 to 46.2)	0.910
Outpatient visits per facility month <5 years, dispensaries	72	2428	−57.5 (−110.2 to −4.9)	0.033	3553	77.11 (44.2 to 110.0)	0.000	3950	−10.08 (−56.0 to 35.8)	0.663

*The beta is the estimated intervention effect controlling for a year dummy and facility-fixed effects.

N, facility months.

We further examined programme effects on structural and process quality of care for targeted services (ANC, PNC and immunisations and delivery care) and non-targeted outpatient services and for delivery care (tables 5 and 6 and online supplemental appendix 5). There was a short-term positive effect on health worker kindness to women during delivery, which was sustained in the longer term. There was also evidence of an improvement in patient satisfaction with patient provider interactions during delivery care in the longer term (by 4 percentage points, p=0.035), whereas no short-term effect had been noted. We found no effect on patient satisfaction with antenatal, postnatal and immunisation services in the short or longer term. An improvement in satisfaction with interpersonal care among non-targeted service users was noted in the short term, but there was no effect in the longer term. While there was no short-term effect on waiting time, we found evidence of a reduction in waiting time due to the programme in the longer term for non-targeted services by around 18 min (p=0.038). Note that none of these effects are significant when correcting for multiple testing (Bonferroni threshold=0.0083).

**Table 5:** Quality of care before and after the introduction of payment for performance

	Intervention			Comparison		
	Baseline	Short term	Long term	Baseline (p value)**	Short term	Long term
**Quality**						
**Targeted services**						
ANC content of care index*	0.53	0.53	0.53	0.49 (0.115)	0.53	0.53
Index of patient satisfaction with interpersonal care for targeted outpatient services (0–1)*	0.72	0.76	0.72	0.70 (0.426)	0.73	0.73
Index of patient satisfaction with interpersonal care during deliveries (0–1)†	0.63	0.69	0.65	0.64 (0.411)	0.69	0.63
Patient assessment of staff kindness during delivery score (1–10)†	7.2	8.0	7.4	7.6 (0.009)	8.0	7.4
Waiting time in minutes*	50.9	59.3	57.8	48.8 (0.793)	50.3	61.4
Consultation time in minutes*	15.8	12.4	13.6	13.6 (0.117)	12.4	13.0
**Non-targeted services**						
Index of patient satisfaction with interpersonal care for non-targeted services*	0.69	0.78	0.71	0.74 (0.007)	0.78	0.76
Waiting time in minutes*	51.4	48.8	42.3	43.7 (0.213)	53.6	57.0
Consultation time in minutes*	13.9	11.4	11.2	13.7 (0.899)	11.1	12.4
**Availability of drugs, supplies and equipment**‡						
Drug availability (%)	60.8	63.9	58.3	65.7 (0.035)	60.7	59.9
Drug stock out (%)	43.1	26.7	42.5	33.5 (0.003)	30.4	41.6
Antimalarials availability (%)§	60.3	69.7	69.4	69.9 (0.021)	59.3	68.9
Antimalarials stock-out (%)§	41.9	29.8	36.3	42.6 (0.886)	40.4	35.0
Delivery care drugs availability (%)¶	39.5	44.8	40.6	41.1 (0.631)	34.6	36.4
Delivery care drugs stock out (%)¶	56.1	35.9	58.0	42.4 (0.003)	46.4	61.1
Medical supplies availability (%)	64.4	66.4	66.5	72.4 (0.032)	66.4	64.2
Medical supplies stock out (%)	39.7	20.8	32.7	29.4 (0.015)	21.8	31.5
Availability of medical equipment (%)	55.0	72.8	61.8	54.9 (0.954)	68.8	56.4

*From exit interviews.

†From household survey.

‡From facility survey.

§ Artemether-Lumefantrine (ALU) and Sulfadoxine-pyrimethamine (SP).

¶Oxytocics and antihypertensives (magnesium sulphate, diazepam, aldomet, nifedipine and hydralazine).

**p values in parenthesis are for the baseline differences between intervention and comparison areas.

ANC, antenatal care; IPT, intermittent presumptive treatment; SP, sulfadoxine–pyrimethamine.

**Table 6:** Effect of payment for performance on quality of care in the short and long term (results from the difference-in-difference analysis)

Outcome variables	N	Short-term effect		Difference between short and long term		Long-term effect	
		Beta* (95% CI)	P value	Beta* (95% CI)	P value	Beta* (95% CI)	P value
**Targeted services**							
ANC content of care index	1090	−0.06 (−0.14 to 0.02)	0.121	0.01 (−0.06 to 0.08)	0.818	−0.01 (−0.09 to 0.06)	0.688
Index of patient satisfaction with interpersonal care for targeted outpatient services	2049	0.04 (−0.01 to 0.09)	0.146	−0.05 (−0.10 to −0.01)	0.025	−0.02 (−0.06 to 0.02)	0.343
Index of patient satisfaction with interpersonal care during deliveries	7648	0.01 (−0.02 to 0.04)	0.505	0.02 (−0.01 to 0.05)	0.127	0.04 (0.00 to 0.07)	0.035
Patient assessment of staff kindness during delivery score (1–10)	7627	0.38 (−0.06 to 0.82)	0.088	0.03 (−0.36 to 0.41)	0.885	0.41 (0.06 to 0.76)	0.019
Waiting time in minutes	2003	6.3 (−16.5 to 29.2)	0.585	−18.1 (−37.8 to 1.6)	0.072	−13.0 (−32.5 to 6.5)	0.190
Consultation time in minutes	1985	−0.8 (−4.5 to 2.9)	0.656	−0.4 (−2.9 to 2.6)	0.759	−0.6 (−1.7 to 0.5)	0.278
** ** **Non-targeted services**							
Index of patient satisfaction with interpersonal care	1779	0.05 (0.01 to 0.10)	0.031	−0.06 (−0.11 to −0.01)	0.030	0.00 (−0.01 to 0.02)	0.850
Waiting time in minutes	1719	−9.3 (−28.3 to 9.6)	0.331	−11.3 (−30.2 to 7.6)	0.238	−18.0 (−35.0 to −0.9)	0.038
Consultation time in minutes	1716	−0.04 (−2.9 to 2.9)	0.977	−2.1 (−4.7 to 0.4)	0.098	−1.9 (−5.0 to 1.0)	0.197
**Availability of drugs, supplies and equipment at the facility**							
Drug availability index (%)	437	8.4 (3.0 to 13.7)	0.002	−4.4 (−9.4 to 0.5)	0.083	3.6 (−1.5 to 9.1)	0.153
Drug stock out index (%)	437	−13.6 (−22.1 to −5.1)	0.002	3.8 (−3.0 to 10.5)	0.273	−9.6 (−16.2 to −3.1)	0.04
Antimalarials availability (%)†	437	20.5 (11.8 to 29.3)	0.000	−8.4 (−18.0 to 1.1)	0.083	11.7 (2.3 to 21.1)	0.015
Antimalarials stock-out (%)†	437	−10.5 (−21.6 to 0.6)	0.064	10.4 (−0.1 to 20.9)	0.052	0.6 (−0.1 to 20.9)	0.922
Delivery care drugs avail (%)‡	437	11.8 (3.8 to 19.8)	0.004	−5.0 (−12.0 to 1.9)	0.154	7.0 (−0.1 to 14.1)	0.052
Delivery care drugs stock out (%)‡	437	−24.7 (−38.4 to −11.0)	0.000	6.0 (−4.7 to 16.8)	0.270	−19.0 (−29.3 to −8.6)	0.001
Medical supplies availability (%)	444	8.3 (0.01 to 16.5)	0.050	2.4 (−3.9 to 8.7)	0.461	10.6 (1.8 to 19.4)	0.018
Medical supplies stock out (%)	428	−13.1 (−23.1 to −3.2)	0.010	0.3 (−7.2 to 7.9)	0.927	−12.3 (−21.5 to −2.8)	0.010
Availability of medical equipment (%)	444	3.8 (−4.9 to 12.6)	0.391	1.7 (−7.7 to 11.1)	0.727	5.7 (−1.9 to 13.2)	0.140

*The beta is the estimated intervention effect controlling for a year dummy, facility-fixed effects, individual-level and household characteristics.

†Artemether-Lumefantrine (ALU) and Sulfadoxine-pyrimethamine (SP).

‡Oxytocics+antihypertensives (magnesium sulfate, diazepam, aldomet, nifedipine, and hydralazine.

ANC, antenatal care; PNC, postnatal care; SP, sulfadoxine–pyrimethamine.

In terms of structural quality, there was evidence of significant improvements in the availability of drugs and medical supplies in the short term, as well as a reduction in their stock out rate. These positive effects reduced in the longer term; the programme effect on overall drug availability was no longer statistically significant, while the reduction in stock-outs was estimated at 9.6 points (p=0.004) in the long term compared with 13.6 points in the short term, with the longer-term effect being driven by a greater increase in stock outs in comparison areas (table 6). Most of the effects on the availability of drugs and medical supplies in the short and long term are also robust to the Bonferroni correction (threshold=0.017), online supplemental appendix 4.

We found evidence of a significant increase in public providers’ adherence to exemptions manifested by a reduced probability of paying out of pocket for deliveries by 5 percentage points (p=0.023) in the short term, increasing to 10 percentage points in the longer term (p<0.001)) (tables 7 and 8). Although the probability of paying for delivery care increased a little in the longer term compared with the short term in the intervention area, the probability of paying rose more substantially in comparison areas (table 7). This effect is robust to the Bonferroni correction (online supplemental appendix 4).

**Table 7:** Cost of services before and after the introduction of payment for performance

Outcome variables	Intervention			Comparison		
	Baseline	Short term	Long term	Baseline (p value)*	Short term	Long term
Probability of paying for ANC (%)	8.1	4.9	5.8	7.5 (0.711)	7.2	4.8
Probability of paying for delivery care (%)	16.5	11.6	12.8	11.9 (0.026)	12.4	18.2
Probability of paying for PNC (%)	6.0	6.2	4.1	7.6 (0.421)	4.0	5.6
Amount paid for ANC, mean US$	0.23	0.28	0.23	0.15 (0.201)	0.08	0.13
Amount paid for delivery, mean US$	1.80	1.83	2.90	2.18 (0.509)	2.48	4.48
Amount paid for PNC, mean US$	0.34	0.32	0.71	0.96 (0.119)	0.13	0.61
Provided a gift for ANC (%)	1.7	2.6	1.3	1.2 (0.403)	0.8	1.3
Provided a gift for delivery (%)	17.4	13.7	15.0	18.8 (0.586)	16.6	20.5
Provided a gift for PNC (%)	7.1	7.1	1.7	4.5 (0.186)	3.9	2.2
Value of gift for ANC, mean US$	0.05	0.10	0.06	0.03 (0.177)	0.09	0.07
Value of gift for delivery, mean US$	0.61	0.52	0.60	0.59 (0.875)	0.67	0.82
Value of gift for PNC, mean US$	0.33	0.33	0.08	0.14 (0.069)	0.19	0.10

*p values in parenthesis are for the baseline differences between intervention and comparison areas.

ANC, antenatal care; PNC, postnatal care.

**Table 8:** Effect of payment for performance on the cost of services in public facilities in the short and long term (results from the difference-in-difference analysis)

Outcome variables	N	Short-term effect		Difference between short and long term		Long-term effect	
		Beta* (95% CI)	P value	Beta* (95% CI)	P value	Beta* (95% CI)	P value
Probability of paying for ANC (%)	7751	−2.7 (−6.0 to 0.6)	0.110	3.0 (−0.1 to 5.9)	0.041	0.3 (−2.9 to 3.4)	0.846
Probability of paying for delivery care (%)	6941	−5.0 (−9.3 to −0.7)	0.023	−5.1 (−9.6 to −0.5)	0.030	−10.3 (−15.2 to −5.4)	0.000
Probability of paying for PNC (%)	2125	2.1 (−3.7 to 7.9)	0.476	−2.8 (−10.8 to 3.0)	0.408	0.1 (−5.5 to 7.0)	0.975
Amount paid for ANC, mean US$ (sd)	7751	0.12 (−0.11 to 0.35)	0.310	−0.13 (−0.38 to 0.12)	0.353	−0.01 (−0.18 to 0.17)	0.971
Amount paid for delivery, mean US$ (sd)	6941	0.19 (−1.17 to 1.55)	0.780	−1.00 (−2.50 to 0.41)	0.186	−0.91 (−2.62 to 0.74)	0.286
Amount paid for PNC, mean US$ (sd)	2125	0.43 (−0.23 to 1.08)	0.202	0.50 (−0.12 to 0.12)	0.465	1.39 (−–0.29 to 3.38)	0.108
Provided a gift for ANC (%)	7786	1.2 (−0.5 to 2.8)	0.158	−1.6 (−3.1 to -0.1)	0.043	−0.4 (−1.9 to 1.1)	0.633
Provided a gift for delivery (%)	6955	−2.8 (−8.2 to 2.6)	0.294	−2.1 (−7.9 to 3.7)	0.484	−4.5 (−10.4 to 1.4)	0.132
Provided a gift for PNC (%)	2163	−0.4 (−6.1 to 5.3)	0.890	−3.0 (−6.8 to 0.7)	0.112	−3.4 (−8.4 to 1.4)	0.163
Value of gift for ANC, mean US$ (sd)	7786	−0.04 (−0.17 to 0.09)	0.569	0.00 (−0.12 to 0.12)	0.979	−0.03 (−0.09 to 0.04)	0.322
Value of gift for delivery, mean US$ (sd)	6955	−0.21 (−0.46 to 0.04)	0.100	−0.05 (−0.32 to 0.21)	0.706	−0.26 (−0.51 to 0.01)	0.050
Value of gift for PNC, mean US$ (sd)	2163	0.00 (−0.32 to 0.33)	0.978	−0.18 (−0.35 to 0.03)	0.048	−0.19 (−0.45 to 0.05)	0.123

*The beta is the estimated intervention effect controlling for a year dummy, facility-fixed effects, individual-level and household characteristics.

ANC, antenatal care; PNC, postnatal care.

#### Heterogeneity of effects

The programme effect on deliveries was significantly pro-poor in both the short and longer term (table 9). The effect was also greater among rural facilities in both the short and longer term. In the short term, the effect was greater among facilities with low baseline performance, but this was no longer the case in the longer term. There were no differential effects of the IPT coverage indicator by local area or facility characteristics (table 9).

**Table 9:** Heterogeneity effect of P4P

Variableˆ	N	Difference-in-difference, short-term effect	Difference-in-difference, long-term effect
		Beta* (p value)	Beta* (p value)
Outcome 1: facility-based delivery coverage			
P4P effect × tercile 1 (poorest population)*	8728	8.91 (0.009)	7.84 (0.009)
P4P effect × tercile 2 (middle wealth)*	8728	5.56 (0.065)	0.82 (0.734)
P4P effect in public facility × tercile 1 (poorest)*	8728	10.8 (0.007)	11.2 (0.005)
P4P effect in public facility × tercile 2 (middle wealth)*	8728	3.2 (0.365)	1.19 (0.722)
P4P effect × public facility	445	4.12 (0.405)	1.50 (0.756)
P4P effect × dispensary facility	445	1.63 (0.723)	1.40 (0.717)
P4P effect × with available utilities	450	−2.99 (0.534)	1.19 (0.774)
P4P effect × low availability of drugs&	435	4.59 (0.317)	2.49 (0.564)
P4P effect × lower baseline performer	450	12.3 (0.005)	4.42 (0.208)
P4P effect × rural facilities	445	9.07 (0.046)	7.48 (0.097)
Outcome 2: IPT2 coverage			
P4P effect × tercile 1 (poorest population)*	7362	0.61 (0.903)	6.07 (0.228)
P4P effect × tercile 2 (middle wealth)*	7362	5.05 (0.263)	7.05 (0.112)
P4P effect × public facility	445	3.62 (0.704)	3.62 (0.679)
P4P effect × dispensary facility	445	−9.14 (0.137)	−6.20 (0.368)
P4P effect × with available utilities	450	−0.99 (0.875)	9.10 (0.201)
P4P effect × low availability of drugs&	435	−1.75 (0.777)	−7.32 (0.283)
P4P effect × lower baseline performer	450	7.62 (0.127)	5.24 (0.389)
P4P effect × rural facilities	445	4.64 (0.574)	−0.89 (0.925)

*All specifications lead to an estimated beta showing percentage point change after controlling for a year dummy, facility-fixed effects and facility-level covariates (availability of utilities and wealth status of the catchment population).

&Availability of drugs include 37 drugs and vaccines, and analysis used a dummy variable classified in each arm separately based on baseline availability distribution (=1 for availability below the median/bottom half and 0, otherwise)

ˆreference category in brackets: public (vs non-public), dispensary (vs health centre and hospital), with electricity and water supply at baseline (vs none), baseline availability of drugs below the median/in bottom half (vs top half), baseline lower performer/below the median (vs higher performer), rural (vs urban district) and poorest/middle wealth (vs least poor).

*Data from household survey.

IPT, intermittent presumptive treatment; P4P, payment for performance.

### Process evaluation findings

#### Programme awareness

During in-depth interviews both district level managers and health workers demonstrated a good understanding of the P4P design components such as objectives, indicators, target setting and bonus distribution formulas. This is in contrast to their more limited knowledge earlier on in the programme, reflecting learning over time. Health worker survey data confirmed increased awareness levels from 85% at 13 months of implementation to 100% at 36 months.

### Programme implementation

#### Bank accounts

When implementation started, a number of facilities had not opened bank accounts, including those in remote areas and faith-based facilities. The health facility survey estimated that 89% of facilities had opened bank accounts by 13 months of implementation, increasing to 96% by 36 months.

#### Bonus payments

Both health workers and managers said there were only small delays in the payments of the bonuses during the first five payment cycles (typically between 1–2 months delay). The payment for cycle 6, however, was 3 months late. As of February 2015, the payment for cycle 7, which was due in September 2014, still had not been made, and informants raised concerns about the delayed payments. The delay led to speculations among some health workers and managers that the scheme might have come to an end:

I thought that it [the scheme] had been stopped. Now I’m surprised they say that it’s still there. I really thought it wasn’t there anymore. (Health worker, Kisarawe district)

#### Data verification

The verification visits conducted by the national Pilot Management Team on a random sample of 25% of facilities once per cycle, ceased from cycle 7. However, district managers continued to conduct verification visits as part of their quarterly routine supportive supervision visits to facilities, a response to the shortage of P4P funds, which prevented managers from conducting separate verification visits as they had previously done. The process of verification which initially varied across districts, was harmonised in 2015, and involved comparing monthly routine health information system reports with patient registers. Health workers felt that P4P had a lasting effect on data compilation, completeness and accuracy. Three of the seven facilities in Kisarawe district had posters to remind health workers of the importance of data on the walls.

### Feedback meetings

Feedback workshops were supposed to be held once per cycle at the district level involving participants from all facilities, to allow reflection of lessons learnt regarding performance across facilities and experience sharing. From cycle 6 onwards the feedback meetings had ceased due to a lack of funds.

### Programme mechanisms

#### Drug procurement

In the first phase of the programme (up to cycle 6), the facility level bonus had been used to procure drugs and supplies with a focus on those drugs needed to deliver incentivised services. However, during the second phase, health workers indicated that delays in receiving funds made it difficult to continue to meet targets in some cases, due to the absence of funds. Antimalarial (sulfadoxine–pyrimethamine, SP), used for the IPT target, was the medication mentioned most often as having been affected by funding delays:

There was a time we ran completely out of SP. If those money [P4P bonus] had come at the correct time, we would have had money to buy SP for the pregnant women. (Health worker Kisarawe district)

The facility survey data showed that in the longer term the availability and stock out rate of IPT had returned to baseline levels.

### Health worker motivation

The delay of funds from cycle 6, and the perception that the P4P intervention had come to an end, affected health worker’s motivation, but not in a uniform manner. At facilities with a low number of staff, the bonus could amount to approximately 50% of a month’s net salary, while it was much lower at facilities with a large number of staff, as the bonus was shared across staff. Staff that received higher bonuses were more likely to voice discontent over the funding delays.

However, a number of respondents suggested that many of the behaviours linked to P4P had become normalised even with the absence of payment.

Before we took it as something monetary, but now we have become used to this as our daily work, we see it as something normal. (…) this is work we are doing out of conscience (…) now P4P is in our blood (…). (Health worker, Bagamoyo district)

There was generally still a sense of hope that even if the funds were delayed, the funding would be forthcoming, and the continued data verification activities by managers supported this:

…for us there is that saying “maybe I’ll get it tomorrow” so we are doing our work. (…). (Health worker, Bagamoyo district)

### Strategies to achieve performance targets

Health workers pointed to a number of ongoing strategies that were used to increase demand among households. Strategies included raising awareness about the dangers of home births and the lack of skills of traditional birth attendants (TBAs). Numerous strategies involving TBAs were mentioned by respondents, including giving TBAs 5000 Tanzanian shillings when they brought a woman to a facility for delivery, warning TBAs that they would be legally responsible if a woman ran into problems while under their care and fining TBAs who assisted in home-based deliveries (though this had not been implemented). However, in several cases, payments to TBAs had ceased with the delayed P4P payments.

## Discussion

This study contributes to the limited evidence examining P4P effects over time, while also trying to explore reasons for variation in effects. Our study found evidence of initial improvements in performance tied to incentivised indicators, coupled with reductions in unincentivised service use in dispensaries. However, our findings generally point to an attenuation of programme effects over time for those indicators that improved in the short term, some improvements in quality of care indicators that did not improve in the short term and the disappearance of negative spill-over effects on unincentivised services. Studies from other LMICs have reported similar short-term increases in targeted outcomes, with sustained effects over time in Mozambique,^15^ and stagnating longer-term effects in Burundi.^35^

The effects of the programme on the rate of institutional deliveries reduced overtime. Although coverage of ANC was maintained, performance on the IPT during ANC target was not sustained over time with stock out rates of IPT increasing over time to baseline levels after an initial reduction. The lack of sustained effect on this indicator is unlikely due to the incentive design (single threshold target), as coverage levels are still below the 80% threshold, but rather due to the funding delays in the longer term due to changes in programme management and funding. Research in Cameroon also reported reduced investment in drugs over time due to delays in incentive payments.^36^

We found that improvements in delivery care utilisation was higher among facilities with lower baseline performance in the short term, however, this differential effect was no longer apparent in the longer term. This is in contrast to US studies which found that facilities with lower baseline performance were more likely to have sustained effects over time.^23^

We found evidence of sustained improvements in kindness during deliveries, and indications of a wider set of improvements in patient experience of delivery care in the longer term. Qualitative data suggests it took time for health workers to fully understand and grasp the programme, which may explain why some of these changes were only observed in the longer term. Research elsewhere also reported that it took time for staff to understand the programme.^37^ The programme effects on process quality are a noteworthy positive spill-over effect, as quality of care indicators were not directly incentivised by the P4P programme in Pwani, unlike many other P4P schemes in sub-Saharan Africa.^38^

Our research suggests that the degree of integration of the P4P scheme within routine systems evolved over time. This was partly tied to adaptations in response to the delayed payment of incentives. For example, managers integrated verification visits within their routine supportive supervision visits, reducing the time impact of the programme on managers and health workers. The lack of longer-term effect on utilisation of non-incentivised services, suggests dispensary staff became more efficient in managing the additional data and reporting requirements over time.

The qualitative data suggests that the introduction of P4P increased extrinsic motivation in the short term, but this happened alongside increased financial management autonomy, and greater relatedness (interactions with managers), with no evidence of harm to intrinsic motivation. Similarly, in Zambia, health workers reported greater job satisfaction linked to enhanced supervision and financial autonomy.^39^ The ongoing benefits of financial autonomy linked to the programme and enhanced supervision, together with hope that funds would eventually arrive, likely sustained motivational effects, despite funding delays. Similarly, in Malawi the goal focus of the programme was motivating in itself, independently of incentives.^40^ However, reductions in performance and motivation linked to uncertainty in obtaining the incentives were reported in Nigeria and Sierra Leone.^41 42^

Our study has a number of limitations. It was not possible to randomly allocate the P4P scheme, and hence we used difference-in-difference methods which relies on assumption that trends in outcomes in intervention and comparison areas would run parallel if the programme had not been implemented. We were, however, able to verify that preintervention trends were similar for a number of outcomes. Second, the measures of non-targeted service use relied on patient register data which were incomplete for some facilities, limiting the available sample for analysis. Third, our assessment of motivational effects are based uniquely on qualitative findings.

Our findings highlight the importance of not just evaluating the effects of P4P at one point in time, but in undertaking ongoing assessment of effects over time. It is clearly important for evaluators to monitor changes in programme design and implementation and how this is related to outcomes, especially as schemes go from pilot to scale, and are taken over by government. This point is true of any intervention that aims to change the way health systems work and health workers behave, and where outcomes are likely to be non-stationary over time. More generally the results demonstrate the limitations of conventional evaluations of cause and effect, and the need to embrace a complex adaptive systems approach to understanding health systems and their response to P4P.^43^ Further research should apply complexity science methods such as system dynamics and agent-based modelling^44^ to increase our understanding of the dynamic, temporal effects of P4P,^45^ and the factors shaping this, so we can build programmes that have sustained effects in the long term.

## Data Availability

Data are available in a public, open access repository. The quantitative data for this paper are made available through Zenodo, DOI: 10.5281/zenodo.5636645, url: https://zenodo.org/record/5636646%23.YanUmtnMK3I.
