# Supplementary material for: Long-term effects of payment for performance on maternal and child health outcomes: evidence from Tanzania
Source: BMJ Glob Health. 2021 Dec 16;6(12):e006409. doi: 10.1136/bmjgh-2021-006409 (PMC8679076; doi:10.1136/bmjgh-2021-006409)
Supplement: Supplementary data [file bmjgh-2021-006409supp001.pdf]

Supplementary Material – Appendices

Appendix 1: Sampling Strategy for Impact Evaluation

The health facility was the primary sampling unit. Facilities were sampled from those that were eligible to participate in the P4P scheme (they offered reproductive and child health services and had submitted a one year backlog of HMIS data, enabling performance targets to be measured). All eligible hospitals (n = 6) and health centres (n = 16) from the intervention districts were included in the sample along with all eligible non-public dispensaries (n = 11). An equivalent number of facilities in control areas were sampled by level of care. Public dispensaries were sampled at random with probability proportional to the number of public dispensaries in a given district (n = 42). In control areas, hospitals and health centres were sampled to match as closely as possible with selected intervention facilities in terms of annual outpatient care visits and staffing levels. A total of 75 health facilities were sampled from intervention districts, and 75 were sampled from control districts. In Pwani region, 46% of all facilities in the region were included in the sample.

The aim of the sampling procedure for the selection of health facilities was to seek district representation, while for the health worker survey it was to obtain the views, attitudes, and perceptions of at least one health worker per facility. No sample size calculation was therefore carried out. In dispensaries, one health worker will be interviewed. If more than one health worker is on duty, preference will be given to someone other than the in-charge to avoid overburdening them with questions (as they will be interviewed for the facility survey). In health centres and hospitals, two health workers will be interviewed. The health workers will be selected at random from those who are on duty at the facility on the day the interviewers are present.

For the exit and household surveys, the sample size calculation was based on the formula by Hayes and Bennett, 1999, adjusted for the cluster design of the study at the facility level. We estimated the size needed to detect a 17% reduction in waiting time from 114 minutes (SD 66) to 95 minutes, with a k value of 0.25, 80% power and a significance level at of 5% (two tailed test). The estimated sample size was 10 exit interviews per facility, equivalent to a total of 750 interviews in intervention and control areas respectively. A balance in the number of interviews between antenatal, postnatal clients and non-targeted services will be sought.

Exit interview patients will be approached by interviewers upon entry to the health facility and asked a series of screening questions to check their eligibility. Eligible patients will then be asked for their informed consent to participate in the study. This process will be repeated until the required number of eligible consenting respondents has been attained. Participants will then be monitored by the interviewers from their time of arrival at the facility until their time of departure, and the waiting and consultation times will be measured using a stopwatch. The cadre of the provider seen by the woman/child will also be recorded by the interviewer. The survey tool will be administered to patients upon completion of their consultation in a quiet location within the facility, at distance from providers and other patients.

For the household survey, we estimated that the required sample size to detect an 11 percentage point increase in institutional deliveries (from 50 to 61%), with k value of 0.25, 90% power, and a significance level at of 5% (two tailed test), and a 90% response rate, was 20 households per cluster, equivalent to 1,500 women per study arm. The following process was followed to identify eligible households. First, villages were sampled from the facility catchment area; for all dispensaries, the village where the facility is located will be selected by the research team; for health centres and hospitals, two villages will be selected at random from all villages lying within the ward where the facility is located. Second, all hamlets (comprising approximately 100 households) within this village/these villages, and located within the catchment area of the facility will be identified; a random sample of four of these hamlets will then be selected. In the case of dispensaries, all four hamlets will reside within the selected village. In the case of health centres and hospitals, two hamlets will be sampled from each village. Third, five households will be sampled from each of the selected hamlets, amounting to a total of 20 households within each facility's catchment area; households will be selected at random from the selected hamlets using a modified Expanded Programme of Immunisation (EPI) type sampling scheme that ensures an equal chance of any household being selected.

Appendix 2: Regression Models

P4P effects were modelled as function of facility fixed effects (γ_j), year fixed effects (δ_t) through survey waves, and a random error term (ε_{ijt}) as shown in the equation below.

$$Y_{ijt} = \alpha_1 + \beta_1(P4P_j \times T_t^1) + \beta_2(P4P_j \times T_t^2) + \delta_1 T_t^1 + \delta_2 T_t^2 + \delta_3 X_{ijt} + \gamma_j + \varepsilon_{ijt} \quad (1)$$

where Y_{ijt} is the outcome of interest of individual *i* in facility *j*'s catchment areas and at time *t*. We defined $P4P_j$ =1 for facilities implementing P4P and zero for control facilities. Time variables were defined as T_t^1=1 at the time of midline survey (January-February 2013) and zero otherwise, T_t^2=1 at the time of the endline (January- February 2015) and zero otherwise. Our coefficients of interest include β_1 which captured the short-term effect, and β_2 for long-term effect. In the analysis of women's and patients' outcomes, we controlled for individual characteristics (education, religion, marital status, occupation, age, number of pregnancies) and household characteristics (insurance status, number of household members, household head education, and wealth based on ownership of household assets and housing particulars) denoted by X_{ijt}. In the regression model, standard errors were clustered at the facility level, or the facility catchment area.

To estimate the difference between the short-term to long-term effects, we estimated the following equation:

$$Y_{ijt} = \alpha_2 + \beta_2(P4P_j \times T_t^2) + \beta_3(P4P_j \times T_t^3) + \delta_4 T_t^2 + \delta_5 T_t^3 + \delta_6 X_{ijt} + \gamma_j + \varepsilon_{ijt} \quad (2)$$

Where the coefficient estimate β_2 is the difference in the change in outcome between the short and the long term, while the coefficient estimate β_3 is equal to β_1 in equation 1. T_t^3=1 if the observation belonged to any period after the introduction of P4P.

We further estimated the heterogeneity of P4P effects across local area characteristics (wealth status, rural/urban location) and characteristics of facilities (level of care, ownership, baseline performance) [29].

$$Y_{ijt} = \alpha_3 + \beta_4(P4P_j \times T_t^1) + \beta_5(P4P_j \times T_t^2) + \beta_6(P4P_j \times T_t^1 \times C_j) + \beta_7(P4P_j \times T_t^2 \times C_j) + \beta_8(C_j \times T_t^1) + \beta_9(C_j \times T_t^2) + \delta_7 T_t^1 + \delta_8 T_t^2 + \delta_9 X_{ijt} + \gamma_j + \varepsilon_{ijt} \quad (3)$$

Equation 3 includes a three-way interaction term to estimate differential effects; the interactions between the P4P intervention, a time variable, and an indicator variable of facility/ local area characteristic (C_j) to capture the subgroup/ differential effects. We also controlled for time-varying facility-level covariates X_{ijt} (availability of electricity and water supply, and the mean wealth index for households sampled in the catchment area of the facility) as potential confounding factors. Thus, β_6 and β_7 were coefficients of interest capturing the differential effects of P4P in the short term and long term, respectively.

Appendix 3: Analysis of pre-trends in household and facility survey data

The difference-in-difference empirical strategy rests on the parallel trends assumption – that is, in the absence of the payment for performance (P4P) intervention trends in outcomes do not differ between intervention and comparison areas. This assumption can never be formally tested. However, we are able to provide supportive evidence by examining pre-trends – trends prior to the introduction of P4P – for those outcomes that we are able to exploit the longitudinal nature of the data.

Outcomes in household data

The household survey interviewed women who had given birth in the past one year and collected data on the month and year of childbirth. We are therefore able to generate longitudinal measures for outcomes related to care given at the time of childbirth. These outcomes include: proportion of women who gave birth in a health facility; proportion of women who had a caesarean section; proportion of women who breastfeed within one hour of birth; and proportion of women who had to pay for delivery care. Childbirth is a single event that occurs at a particular moment in time. The same is not true for other outcomes, such as those related to antenatal care.

We first present descriptive evidence showing that the four outcomes of interest at baseline are similar in terms of both the level and the pre-trend (Figure A). More formally, we test for a divergence in pre-trends by estimating the following regression:

$$y_{ijt} = \beta t + \gamma D_j t + \theta_j + X_{ij} + \varepsilon_{ijt} \quad (1)$$

where y_{ijt} is the outcome of individual *i* in facility catchment *j* in month t, *t* represents the month since the start of the data period, β is the monthly trend is the comparison districts, γ is the difference in the trend in the intervention districts, θ_j are facility fixed-effects, X_{ij} are characteristics of the woman, and ε_{ijt} is the error term.

We were unable to reject the null hypothesis of equal pre-trends for any of the outcomes. The estimated values of γ were: i) facility based delivery coefficient of -0·0001 (95% CI -0·0004, 0·0002; p value=0·580); c-section coefficient of -0·00002 (95% CI -0·0002, 0·0002; p value=0·893); early breast-feeding coefficient of 0·0001 (95% CI -0·0002, 0·0004; p value=0·683); and pay for delivery coefficient -0·0001 (95% CI -0·0001, 0·0002; p value=0·618). Taken together, these findings provide reassuring evidence that the introduction of the P4P scheme is orthogonal to the error term.

Figure A: Differences in pre-trends

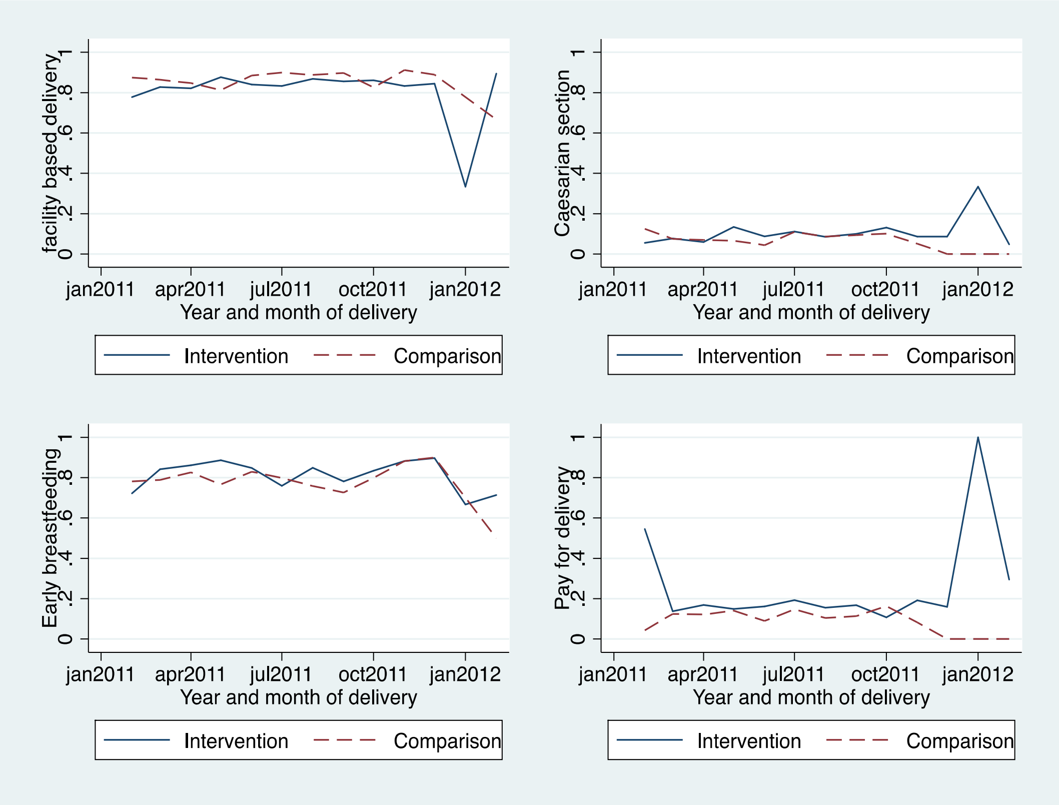

Outcomes in facility data

We tested for divergent pre-trends for utilisation outcomes from patient registers in a similar manner. As above, the coefficient on the interaction between the P4P intervention variable and the linear monthly time trend shows the difference in the pre-trend and is reported here below with the corresponding p value. There is no evidence of divergent pre-trends for the majority of outcomes studied. However, the findings do suggest that trends for family planning visits and outpatient visits under five were rising in the intervention group relative to the comparison group prior to the introduction of P4P. Impact results related to these two outcomes should therefore be interpreted with particular caution.   When trends are rising faster in the intervention group we are concerned with potential positive bias – eg. a spurious finding of a positive impact. For family planning visits and outpatient visits under five we found no effect. This may be a spurious finding and in fact there was a negative effect, which simply reinforces our results from the dispensary only analysis.

Normal deliveries: 0·002 (p=0·979)
Normal deliveries (excluding hospitals): -0·036 (p=0·651)
Polio: 0.576 (p=0·235)
Measles: -0.121 (p=0·454)
DPT: 0.114 (p=0·771)
Family planning visits: 1·54 (p=0·039)
ANC visits: -0·441 (p=0·297)

ANC first visits: -0·124 (p=0·435)
Outpatient visits under five: 5·474 (p=0·037)
Outpatient visits under five (dispensaries only): 2·033 (p=0·306)
Outpatient visits over five: 5·067 (p=0·227)
Outpatient visits over five (dispensaries only): -2·565 (p=0·419)

Appendix 4 Corrections for multiple hypothesis testing.

To take the multiple testing into account, we correct the p-values by hypothesis using the Bonferroni correction (the p-value threshold for statistical significance at the 5% level becomes equal to 0.05/(number of tests)).

We have 14 main hypotheses. Here is the list of hypotheses, the tables where the hypotheses are tested, the number of statistical tests made per hypothesis and the Bonferroni threshold corresponding to the 0.05 level of significance:

1. P4P had a short term-effect on targeted services (Table 5, upper panel, 9 tests, Bonferroni threshold = 0.0055);
2. P4P had a long term-effect on targeted services (Table 5, upper panel, 9 tests, Bonferroni threshold = 0.0055);
3. P4P had a short term-effect on non-targeted aspects of targeted services (Table 5, lower panel, 3 tests, Bonferroni threshold = 0.017);
4. P4P had a long term-effect on non-targeted aspects of targeted services (Table 5, lower panel, 3 tests, Bonferroni threshold = 0.017);
5. P4P had a short term-effect on non-targeted services (Table 6, 4 tests, Bonferroni threshold = 0.0125);
6. P4P had a long term-effect on non-targeted services (Table 6, 4 tests, Bonferroni threshold = 0.0125);
7. P4P had a short term-effect on the quality of targeted services (Table 7, upper panel, 6 tests, Bonferroni threshold = 0.0083);
8. P4P had a long term-effect on the quality of targeted services (Table 7, upper panel, 6 tests, Bonferroni threshold = 0.0083);
9. P4P had a short term-effect on the quality of non-targeted services (Table 7, middle panel, 3 tests, Bonferroni threshold = 0.017);
10. P4P had a long term-effect on the quality of non-targeted services (Table 7, middle panel, 3 tests, Bonferroni threshold = 0.017);
11. P4P had a short term-effect on the availability of drugs, supplies and equipment (Table 7, lower panel, 9 tests, Bonferroni threshold = 0.0055);
12. P4P had a long term-effect on the availability of drugs, supplies and equipment (Table 7, lower panel, 9 tests, Bonferroni threshold = 0.0055);
13. P4P had a short term-effect on the cost of care (Table 8, 12 tests, Bonferroni threshold = 0.0042);
14. P4P had a long term-effect on the cost of care (Table 8, 12 tests, Bonferroni threshold = 0.0042).

Appendix 5: Graphical representation of results for significant variables in long term effects model

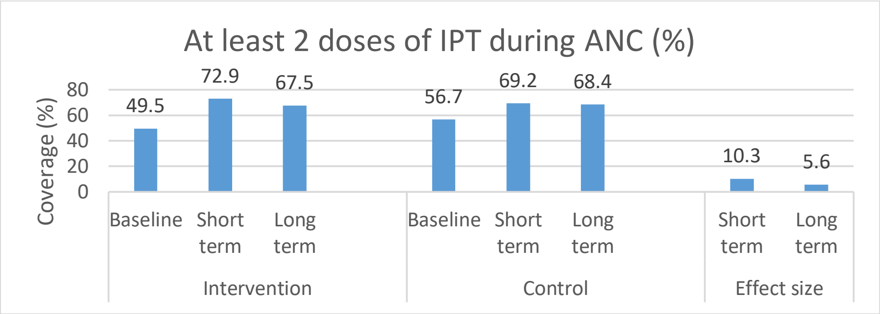

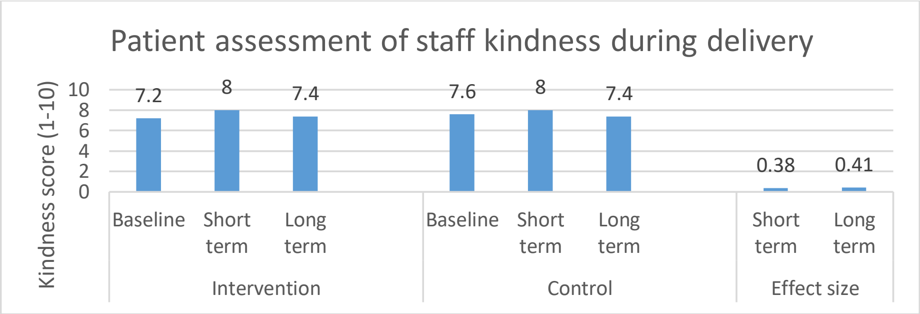

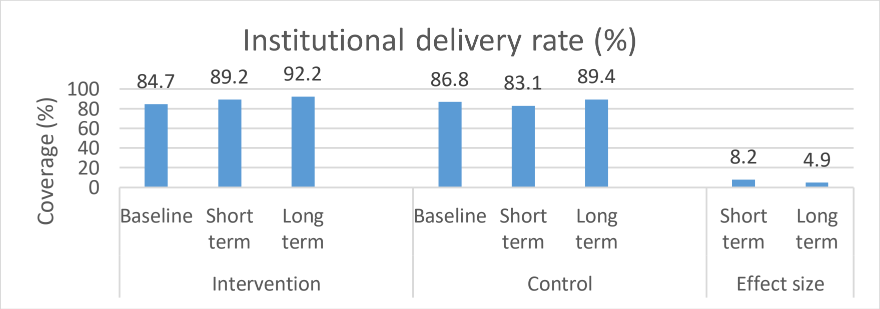

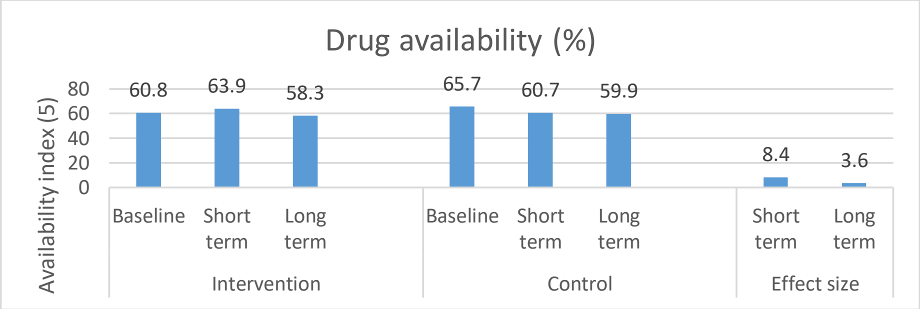

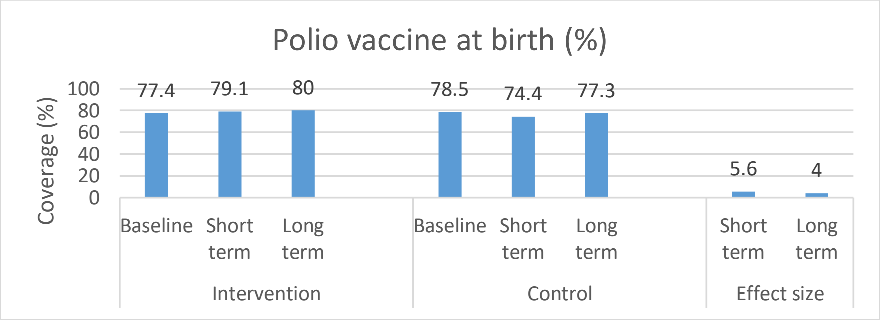
Polio vaccine at birth (%)
Coverage (%)
100
80
60
40
20
0
77.4
79.1
80
78.5
74.4
77.3
5.6
4
Baseline
Short term
Long term
Baseline
Short term
Long term
Short term
Long term
Intervention
Control
Effect size

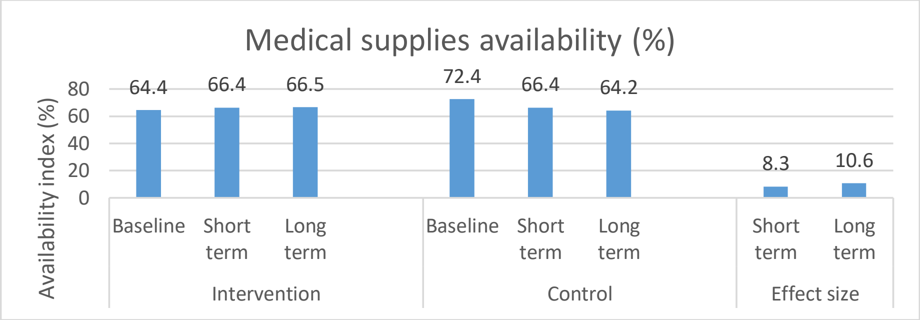
Medical supplies availability (%)
Availability index (%)
80
60
40
20
0
64.4
66.4
66.5
72.4
66.4
64.2
8.3
10.6
Baseline
Short term
Long term
Baseline
Short term
Long term
Short term
Long term
Intervention
Control
Effect size

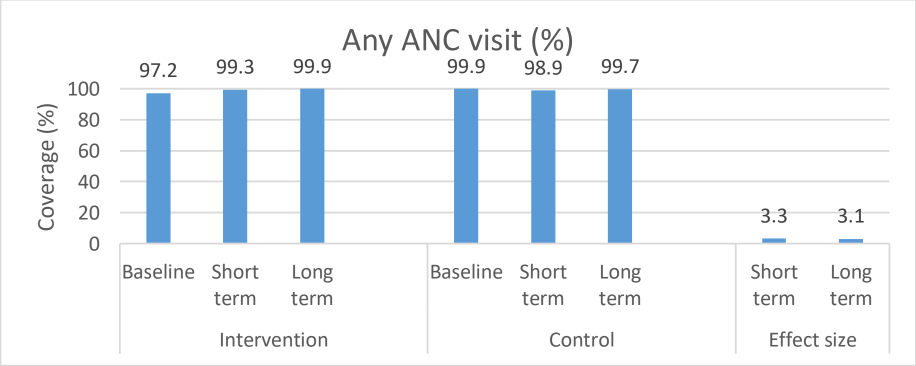
Any ANC visit (%)
Coverage (%)
100
80
60
40
20
0
97.2
99.3
99.9
99.9
98.9
99.7
3.3
3.1
Baseline
Short term
Long term
Baseline
Short term
Long term
Short term
Long term
Intervention
Control
Effect size

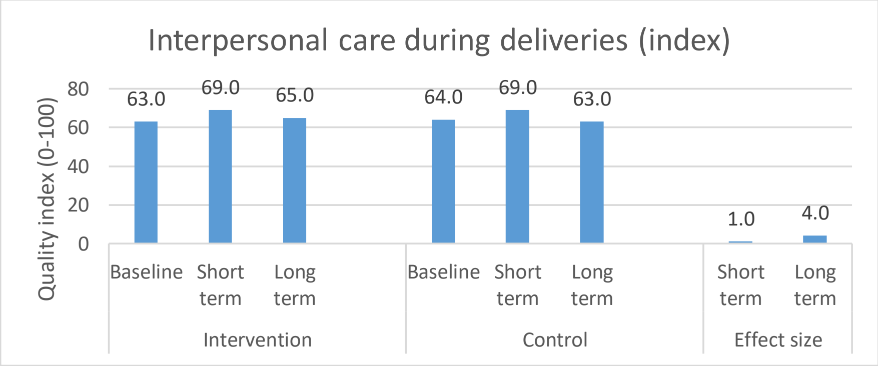
Interpersonal care during deliveries (index)
Quality index (0-100)
80
60
40
20
0
63.0
69.0
65.0
64.0
69.0
63.0
1.0
4.0
Baseline
Short term
Long term
Baseline
Short term
Long term
Short term
Long term
Intervention
Control
Effect size

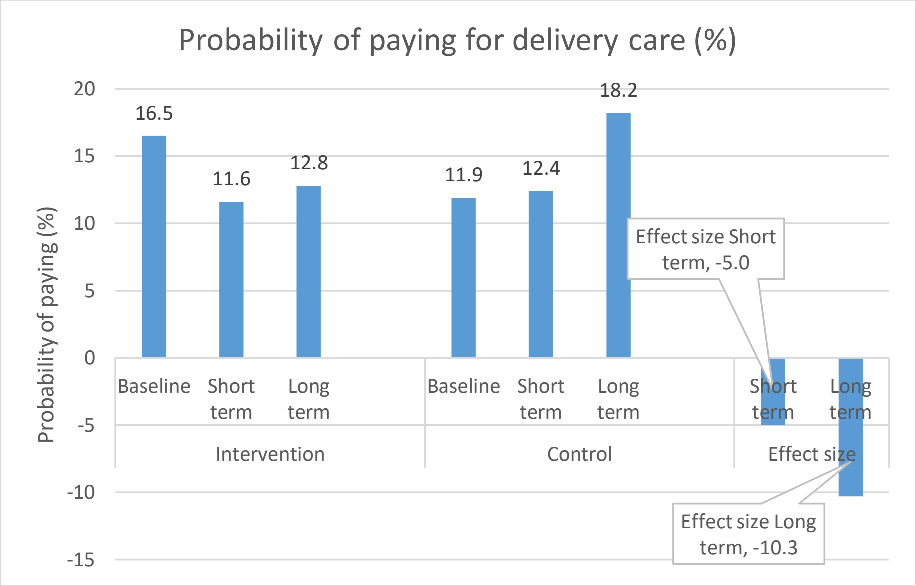
Probability of paying for delivery care (%)
Probability of paying (%)
20
15
10
5
0
-5
-10
-15
16.5
11.6
12.8
11.9
12.4
18.2
Baseline
Short term
Long term
Baseline
Short term
Long term
Short term
Long term
Intervention
Control
Effect size
Effect size Short term, -5.0
Effect size Long term, -10.3

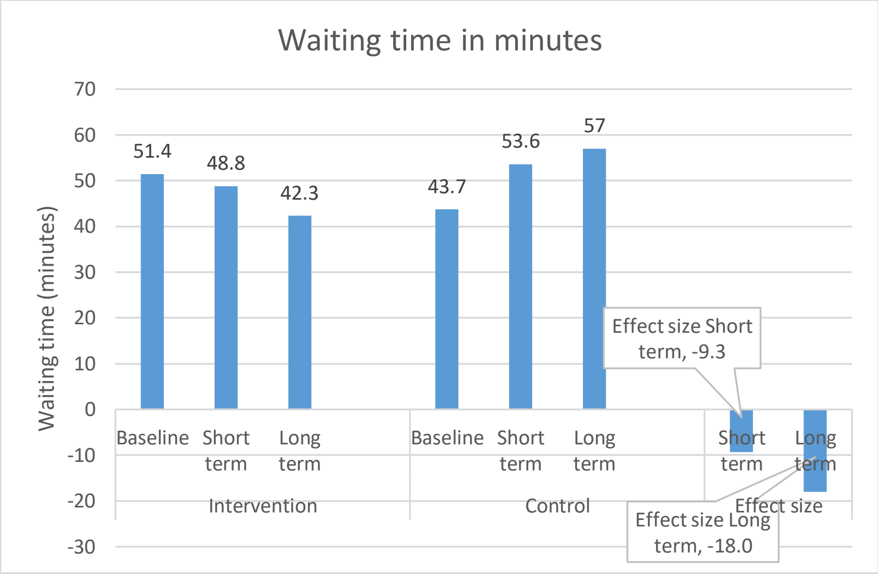
Waiting time in minutes
Waiting time (minutes)
70
60
50
40
30
20
10
0
-10
-20
-30
51.4
48.8
42.3
43.7
53.6
57
Baseline
Short term
Long term
Baseline
Short term
Long term
Short term
Long term
Intervention
Control
Effect size
Effect size Short term, -9.3
Effect size Long term, -18.0
